# Infective Endocarditis Caused by Rhodococcus equi in an Immunocompetent Patient

**DOI:** 10.7759/cureus.7829

**Published:** 2020-04-25

**Authors:** Rony Shah, Linda C Klumpp, Nishant Nerella, Gustine Liu-Young, Jeffrey Jordan

**Affiliations:** 1 Internal Medicine, Citrus Memorial Hospital, Inverness, USA; 2 Cardiology, Citrus Memorial Hospital, Inverness, USA; 3 Infectious Disease, Citrus Memorial Hospital, Inverness, USA; 4 Infectious Disease, Bayfront Seven Rivers Hospital, Crystal River, USA

**Keywords:** infective endocarditis, rhodococcus equi, rhodococcus equi endocarditis, immunocompetent

## Abstract

Rhodococcus equi (R. equi) is a rare zoonotic organism that is found in the feces of grazing animals and in farm soil. It typically causes pulmonary disease, but it can also cause extrapulmonary disease. Immunocompromised patients are at a higher risk of developing the infection, but it has been reported in individuals with competent immune system as well. We present a unique case of infectious endocarditis (IE) due to a R. equi infection in an immunocompetent patient.

A 77-year-old male with a history of coronary artery disease, prior myocardial infarction, systolic heart failure, hypertension, hyperlipidemia, aortic stenosis, and benign prostatic hypertrophy was evaluated by cardiothoracic surgery for coronary and valvular heart disease. His transesophageal echocardiogram and cardiac catheterization demonstrated severe aortic stenosis and multivessel coronary artery disease. The patient underwent coronary artery bypass grafting and simultaneous aortic valve replacement. Intraoperatively, there was exudative material covering his aortic valve, which was sent for tissue culture. Tissue culture was positive for R. equi and Enterococcus faecium.

R. equi endocarditis is a rare presentation of this organism. R. equi endocarditis is a very challenging diagnosis due to its varying presentation compared to typical IE. Detailed history taking and physical exam are extremely important to determine if further evaluation is needed. Prolonged oral and intravenous antibiotics are recommended for effective treatment.

## Introduction

Rhodococcus equi (R. equi) is an uncommon human pathogen present in the feces of many grazing animals and in farm soil [1]. It typically presents with pulmonary disease such as pneumonia and lung abscess in an immunocompromised patient [1]. R. equi infections can be fatal in immunocompromised patients [2]. However, it has been reported in patients with competent immune system [3]. Less than 15% of reported cases occur in immunocompetent patients [4]. The mechanism of human immunological response to this pathogen remains largely unknown which makes it difficult to identify predisposing factors [1]. The diagnosis of R. equi is challenging because it is similar to other bacterial pathogens, such as diptheroids, Mycobacterium, and Nocardia species [5]. Standard treatment regimen for R. equi has not been established due to the rarity of the disease, although a combination of antibiotics has been recommended [3]. We present a unique case of infectious endocarditis (IE) due to a R. equi infection in an immunocompetent patient.

## Case presentation

**Figure 1:**
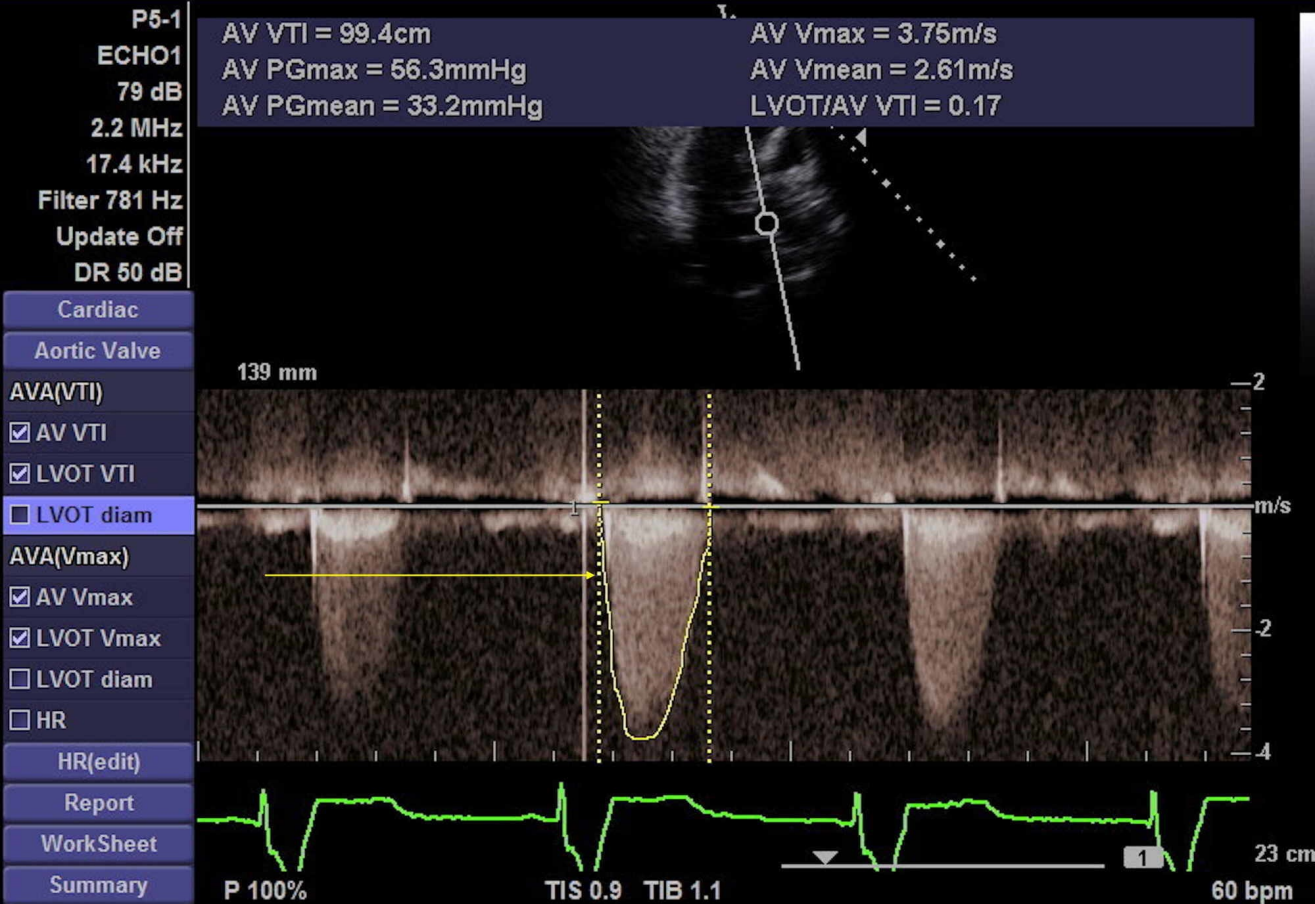
Transthoracic Echocardiogram (TTE) TTE shows moderate to severe aortic stenosis

A 77-year-old male was evaluated by cardiothoracic surgery for coronary and valvular heart disease. He has a past medical history of coronary artery disease, prior myocardial infarction, systolic heart failure, hypertension, hyperlipidemia, aortic stenosis, and benign prostatic hypertrophy. Transthoracic echocardiogram (TTE) demonstrated an ejection fraction of 40%, with evidence of severe aortic stenosis (Figure 1). Cardiac catheterization demonstrated left main coronary artery 95% stenosis, left anterior descending artery 90% stenosis, left circumflex 95% stenosis, and second obtuse marginal artery 95% stenosis. The patient underwent two-vessel (left anterior descending artery, first obtuse marginal artery) coronary artery bypass graftings. Left internal mammary artery and a segment of saphenous vein were used for the procedure. He underwent elective aortic valve replacement as well due to severe valvular and annular calcific stenosis. Aortic valve was replaced with a 23-mm Magna pericardial tissue heart valve (Edwards Lifesciences, Irvine, CA). Intraoperatively, there was exudative material covering his aortic valve, and it was sent for a tissue culture. Tissue culture was positive for R. equi and Enterococcus faecium. The patient was started on intravenous (IV) 1.25 g vancomycin, azithromycin 500 mg daily, and rifampin 300 mg for six weeks per infectious disease recommendations. The patient completed the medication regimen and was stable at outpatient follow-up with no evidence of infection.

## Discussion

R. equi is a rare zoonotic organism, aerobic gram-positive coccobacillary organism, which is commonly found on dry surface soil of farming and livestock properties. The first human case was reported in 1967 when a young male patient on corticosteroid and 6-mercaptopurine for autoimmune hepatitis developed cavitary pneumonia [1]. It is acquired through inhalation, ingestion, or inoculation of a wound. Our patient lives in an area close to horse farms which is his potential source of exposure.

The majority of infections are reported in immunocompromised individuals such as organ transplant recipients, AIDS patients (CD4<100), individuals on chemotherapy for malignancy, and those on corticosteroids or anti-TNF antibody therapy. Infections do occur in immunocompetent patients. Our patient was immunocompetent with an extensive cardiac history. R. equi most commonly causes pulmonary infections. However, it can disseminate and cause disease in any human tissue. R. equi has been cultured from a variety of human tissues such as heart valves, cerebrospinal fluid, skin, lymph nodes, bone, pericardium, and peritoneal fluid causing a wide spectrum of diseases [1]. 

Zoonotic bacteria that commonly cause IE are Coxiella burnetii, Bartonella henselae, and Chlamydia psittaci. After literature review, we identified only two reported cases of IE caused by R. equi in an immunocompetent patient. One of the case reports discussed an indolent, slowly progressing course without any vascular or immunological complications but with mild valvular damage [6]. The other case report discussed R. equi endocarditis with extremely rare complications associated with IE [2]. Our patient did not present with any IE manifestations. It was an incidental finding intraoperatively during cardiac surgery which was not demonstrated on TTE and transesophageal echocardiography. Diagnosing R. equi endocarditis is very challenging due to its varying presentation compared to typical IE presentation.

Treatment of R. equi is complicated by antimicrobial resistance, treatment failures, and clinical relapses. Multidrug therapy of at least two or three active agents is more effective compared to monotherapy based on available evidence [1]. R. equi is usually susceptible to macrolides, rifampicin, aminoglycosides, fluoroquinolones, glycopeptides, and imipenem [5]. We treated our patient with IV vancomycin, oral azithromycin, and oral rifampin for six weeks. Prolonged duration of IV and oral antimicrobial therapy is recommended for effective treatment [1]. Most patients require IV antibiotics for more than two weeks for clinical improvement to be observed [2]. Rhodococcus species are facultative intracellular bacteria which may persist and replicate within macrophages. This allows for frequent relapse despite antimicrobial treatment [1].

## Conclusions

R. equi is a rare opportunistic pathogen commonly found in animals. Although majority of the infections occur in immunocompromised patients, infections have been reported in patients with a normal immune system. This organism commonly causes pulmonary infections but it can cause extrapulmonary disease as well. Diagnosis and treatment of R. equi can be challenging for physicians due to the rarity of the disease. R. equi endocarditis in an immunocompetent patient is a rare presentation of this organism with limited cases reported in literature.
